# Association Between Workplace Absenteeism and Alcohol Use Disorder From the National Survey on Drug Use and Health, 2015-2019

**DOI:** 10.1001/jamanetworkopen.2022.2954

**Published:** 2022-03-17

**Authors:** Ian C. Parsley, Ann Marie Dale, Sherri L. Fisher, Carrie M. Mintz, Sarah M. Hartz, Bradley A. Evanoff, Laura J. Bierut

**Affiliations:** 1 Department of Psychiatry, Washington University School of Medicine, St Louis, Missouri; 2 Department of Medicine, Washington University School of Medicine, St Louis, Missouri

## Abstract

**Importance:**

Alcohol use disorder (AUD) is common and associated with increased morbidity. The degree to which AUD currently factors into workplace absenteeism needs further characterization in the US.

**Objective:**

To examine the association between AUD and workplace absenteeism in a nationally representative sample.

**Design, Setting, and Participants:**

This cross-sectional study used data from a nationally representative sample of noninstitutionalized US residents from the 2015-2019 National Survey on Drug Use and Health to examine the association of AUD with workplace absenteeism. Eligible respondents were aged 18 years and older who reported full-time employment. Data were analyzed from March to September 2021.

**Main Outcomes and Measures:**

Primary outcomes were markers of workplace absenteeism as defined by the number of days missed from work because of illness or injury and days skipped from work in the last 30 days. Descriptive statistics, prevalence ratios, and logistic regression analyses were performed to assess the association between AUD and absenteeism.

**Results:**

A total of 110 701 adults aged 18 years and older reported current full-time employment (58 948 [53.2%] men, 51 753 [46.8%] women; 12 776 [11.5%] Black, 18 096 [16.3%] Hispanic, and 69 506 [62.8%] White respondents). Weighted prevalence of AUD in this sample of working adults was 9.3% (95% CI, 9.0%-9.5%); 6.2% (95% CI, 6.0%-6.4%) of respondents met criteria for mild AUD, 1.9% (95% CI, 1.7%-2.0%) for moderate AUD, and 1.2% (95% CI, 1.1%-1.3%) for severe AUD. Mean days missed from work annually increased in a stepwise fashion with increasing AUD severity (no AUD, 13.0 days; 95% CI, 12.7-13.2 days; mild AUD, 17.7 days; 95% CI, 16.4-19.1 days; moderate AUD, 23.6 days; 95% CI, 21.5-25.7 days; severe AUD, 32.3 days; 95% CI, 27.5-37.0 days). People with AUD represented 9.3% of the full-time workforce and contributed to 14.1% of total reported workplace absences.

**Conclusions and Relevance:**

In this cross-sectional study, AUD was disproportionately associated with an increased prevalence of workplace absenteeism, with individuals with AUD contributing over 232 million missed workdays annually. These results provide economic incentive for increased investment in AUD prevention and treatment, both for employers and policy makers.

## Key Points

**Question:**

What is the association between alcohol use disorder (AUD) and workplace absenteeism?

**Findings:**

In a nationally representative, cross-sectional sample of 110 701 full-time employed adults, mean days missed from work were statistically higher among individuals with AUD. The estimated mean number of annual days absent per individual increased in a stepwise fashion with increasing AUD severity.

**Meaning:**

These results suggest that individuals with AUD disproportionately miss work, providing an economic rationale for policy makers and employers to prevent and provide treatment for this diagnosis.

## Teaser

This cross-sectional study using data from a national survey of US adults examines the association of alcohol use disorder with days missed from work.

## Introduction

Alcohol use is highly prevalent in the US. In the 2019 report from the National Survey on Drug Use and Health (NSDUH), 55% of respondents 18 years and older reported using alcohol within the past month,^1^ and alcohol use disorder (AUD) continues to be one of the most prevalent substance use disorders in the US.^2^ In 2019, 9.3% (3.1 million people) of Americans between ages 18 to 25 years and 5.1% (11 million people) of those 26 years and older reported past-year AUD.^1^

Because of its prevalence, the impact of AUD on society is substantial. AUD is one of the leading causes of preventable death in the US; it is estimated that excessive alcohol use results in more than 95 000 deaths annually.^3^ Concerningly, alcohol-associated mortality has increased in recent years, particularly in the setting of the COVID-19 pandemic.^4,5^ In addition to mortality, excessive alcohol use causes significant increased morbidity, both from physical and psychological standpoints.^6,7^ Considering the significant medical consequences, legal complications, and social difficulties, the effects from problematic alcohol use have been estimated to cost the US economy over $249 billion annually.^8^

Given the prevalence of AUD and its impact on morbidity and mortality, it has long been assumed that AUD has a similar negative effect on an individual’s functioning in the workplace. Although there are several ways to study functional impairment in the workplace, a commonly used metric is work absenteeism. Defined as not attending work when otherwise expected to, this outcome captures 1 important aspect of impairment from alcohol use.^9^ Large-scale epidemiologic studies undertaken in Sweden, Denmark, Finland, Australia, and other countries have identified an association between heavier alcohol use, AUD, and increased work absenteeism.^10,11,12,13,14^ Research from the mid-1990s demonstrated that heavy alcohol use was associated with increased workplace absenteeism in the US.^15,16^ A more recent study using 2012 to 2014 national survey data showed an association between AUD and absenteeism, but it was not investigated extensively or examined with current clinical criteria for AUD.^17^

Because of changes in the workplace, the workforce, and alcohol consumption over the last decade, our goal was to examine the current association of AUD with workplace absenteeism in the US. This project builds on earlier findings using data from the NSDUH, a large, nationally representative sample using established diagnostic criteria to define AUD, to examine the association between AUD over the previous 12 months and workplace absenteeism. In addition, we controlled for other factors that could potentially affect the association between AUD and workplace absenteeism (eg, severe psychological distress, history of conduct problems). We asked several questions: what is the prevalence of past 12-month AUD among the full-time workforce in the US? What is the prevalence of absenteeism among full-time workers with AUD? What is the association between AUD and workplace absenteeism?

## Methods

### Study Sample

Data were drawn from the 2015-2019 surveys of the NSDUH. This cross-sectional survey is administered every year by the Substance Abuse and Mental Health Services Administration (SAMHSA), which selects a nationally representative sample of noninstitutionalized individuals 12 years of age and older from all 50 states and the District of Columbia. Individuals without a fixed address, active duty in the military, or living in institutional group quarters (eg, prisons, hospitals) are not included.^18^ The NSDUH includes data on past-year alcohol use behaviors, as well as a broad set of questions on recent employment status and physical and mental well-being. Interviews are administered in person by field workers, and questions regarding potentially sensitive behaviors, including alcohol use, are administered via computer-assisted self-interview to maintain confidentiality. We excluded interviews conducted prior to 2015 because of significant changes in methods of administering questions related to employment status. Our analysis was limited to adults (age 18 years and older) who provided responses to selected employment and alcohol use questions as described below. Weighted interview response rates for the 2015-2019 NSDUH surveys ranged from 64.9% to 69.7%. The RTI International institutional review board reviewed the NSDUH protocol and all participants provided verbal informed consent for data collection, analysis, and publication. Deidentified data were obtained for analysis and no further institutional review board approval was required.

### Definition of AUD

Mild, moderate, and severe AUD diagnoses were generated using questions in NSDUH that mirror *Diagnostic and Statistical Manual of Mental Disorders* (Fifth Edition) (*DSM-5*) criteria. Notably, a decision was made to use *DSM-5* instead of *DSM-IV* criteria due to its clinical relevance and better characterization of functional impairment (definition of AUD in eAppendix and eTable 1, concordance between *DSM-5* AUD and *DSM-IV* alcohol abuse or dependence in eTable 2 in the Supplement).

### Definition of Employment

The variable used to define current employment status was created based on individual responses to questions assessing job status during the week prior to completing the survey. Employed individuals were defined as those who provided positive responses to either “Did you work at a job or business at any time last week?” or “Even though you did not work at any time last week, did you have a job or business?” Among those who were employed, full-time employment status was defined by a positive response to the question, “Do you usually work 35 hours or more per week at all jobs or businesses?” Only full-time workers were included because part-time status was less well-characterized in the data set and because the majority of previous studies assessed full-time employment, which allowed for better comparison with our results.

### Primary Outcome

The primary outcome of interest was workplace absenteeism among full-time workers. This outcome was assessed using 2 separate questions, “During the past 30 days ... how many whole days of work did you miss because you were sick or injured?” and, “During the past 30 days … how many whole days of work did you miss because you just didn’t want to be there?” These questions were asked of all currently employed individuals. These 2 measures of absenteeism are similar to those used in previous studies.^19^ These continuous variables were used to create 2 new categorical variables capturing substantial work absenteeism, defined as missing 3 or more days of work per month due to illness or injury or missing 3 or more days per month due to skipping work.

### Statistical Analysis

SAS version 9.4 (SAS Institute) was used to conduct all analyses. We used survey procedures to account for NSDUH’s complex sampling design. We estimated weighted frequencies for demographic characteristics within our sample and used χ^2^ tests or *t* tests to determine whether demographic characteristics differed by AUD status. Workplace absenteeism was calculated for each category of AUD severity. Annual estimates were calculated by multiplying the mean monthly number of days absent by 12. All of these estimates were calculated using unadjusted models (ie, before controlling for covariates).

To further examine the association between AUD status and work absenteeism, we estimated unadjusted prevalence ratios, unadjusted odds ratios, and adjusted odds ratios. For these analyses, proc surveyfreq and proc surveylogistic were utilized in SAS. Given that prevalence of the primary outcome (workplace absenteeism) was less than 10% throughout the sample, adjusted odds ratios were deemed to be a reasonable approximation of adjusted prevalence ratios.

Covariates were included in logistic regression analyses based on their previously identified potential to factor into the association between AUD and workplace absenteeism. Covariates included age, sex, race and ethnicity (African American or Black, Asian, Hispanic, White, and other [including Native American or Alaska Native, Native Hawaiian or other Pacific Islander, or those identifying as more than 1 race or ethnicity]), and marital status. Additionally, severe psychological distress and prior documented history of conduct problems (ie, tendency toward rule-breaking) were deemed potentially important to consider. Severe psychological distress in the last month was determined from the summation of respondents’ self-reports of recent symptoms and directly mimic the K6 scale, a validated scale commonly used clinically to screen for recent psychological distress by asking respondents to rate the severity of symptoms in various psychiatric domains.^20^ A positive response for severe psychological distress is defined as a total score greater than 13 (on a Kessler 6-item scale, range 0 to 24), which has been shown to be highly correlated with diagnosis of serious mental illness.^21^ A history of conduct problems was defined as endorsement of any of the following 3 behaviors in the last 12 months: selling illegal drugs, stealing more than $50 worth of items, or attacking someone with the intent of seriously hurting them.

Covariates were included in the adjusted logistic regression model based on analyses performed to assess both the presence of interaction between AUD status and each covariate as well as evidence of the covariate confounding the association of AUD status with absenteeism. Interaction terms were assessed both through visual inspection of stratum-specific associations and through formal statistical analyses, including Breslow-Day and Pearson goodness-of-fit tests. No clear interactions were identified. Age, sex, and race and ethnicity were included in the analyses given the known differences in prevalence of AUD by these variables. History of conduct problems and severe psychological distress were included in the final models based on their evidence of significant contribution to the model. Marital status, income, and education level were not deemed to contribute significantly and were dropped from further analyses. *P* < .05 in 2-sided tests and 95% CIs were used to determine significance.

## Results

### Sample Demographics

Of the 214 505 total respondents, 110 701 respondents met inclusion criteria of being 18 years or older and being employed full-time; this group was used for further analyses (58 948 [53.2%] men, 51 753 [46.8%] women; 12 776 [11.5%] Black, 18 096 [16.3%] Hispanic, and 69 506 [62.8%] White respondents). Missing responses to employment status were negligible and their demographics did not differ significantly from those included in the analysis (eFigure in the Supplement). There were no missing data for sex, age, race and ethnicity, income, and recent severe psychological distress variables. For the history of conduct problems, 142 responses were missing, which was considered negligible. Weighted prevalence of any past-year AUD among full-time workers was 9.3% (95% CI, 9.0%-9.5%); 6.2% (95% CI, 6.0%-6.4%) of respondents met criteria for mild AUD, 1.9% (95% CI, 1.7%-2.0%) for moderate AUD, and 1.2% (95% CI, 1.1%-1.3%) for severe AUD. AUD was relatively more prevalent among men (weighted percentage of respondents in overall sample: 65.6% vs no AUD, 55.5%), younger respondents (ages 18-29 years, 35.3% vs 19.9%), and respondents identifying as White (66.2% vs 63.8%) and Hispanic (17.1% vs 16.2%) (Table 1).

**Table 1.** Demographic Variables by AUD Status Among Adult Full-Time Workers

Characteristic	Respondents, No. (%)				*P* value
	No AUD		AUD		
	Raw	Weighted	Raw	Weighted	
Participants	98 506 (89.0)	109 761 112 (90.7)	12 195 (11.0)	11 215 870 (9.3)	NA
Sex					
Men	51 531 (52.3)	60 874 280 (55.5)	7417 (60.8)	7 359 214 (65.6)	<.001
Women	46 975 (47.7)	48 886 832 (44.5)	4778 (39.2)	3 856 656 (34.4)	
Age, y					
18-29	35 656 (36.2)	21 792 189 (19.9)	6403 (52.5)	3 954 432 (35.3)	<.001
30-49	47 995 (48.7)	50 036 085 (45.6)	4915 (40.3)	4 955 505 (44.2)	
≥50	14 855 (15.1)	37 932 838 (34.6)	877 (7.2)	2 305 932 (20.6)	
Race and ethnicity					
African American or Black	11 572 (11.7)	12 604 460 (11.5)	1204 (9.9)	1 134 445 (10.1)	<.001
Asian	4686 (4.8)	6 810 869 (6.2)	397 (3.3)	427 073 (3.8)	
Hispanic	16 074 (16.3)	17 765 126 (16.2)	2022 (16.6)	1 920 621 (17.1)	
White	61 660 (62.6)	69 980 863 (63.8)	7846 (64.3)	7 430 242 (66.2)	
Other^a^	4514 (4.6)	2 599 794 (2.4)	726 (6.0)	303 488 (2.7)	
Past-month serious psychological distress					
No	93 570 (95.0)	105 728 269 (96.3)	10 401 (85.3)	9 903 759 (88.3)	<.001
Yes	4936 (5.0)	4 032 843 (3.7)	1794 (14.7)	42 478 (11.7)	
Past-year history of conduct problems					
No	96 383 (97.8)	108 072 621 (98.5)	11 127 (91.2)	10 524 027 (93.8)	<.001
Yes	2123 (2.2)	1 688 491 (1.6)	1068 (8.8)	691 842 (6.2)	

Abbreviations: AUD, alcohol use disorder; NA, not applicable.

^a^ Other racial category included Native American or Alaska Native, Native Hawaiian or other Pacific Islander, or those identifying as more than 1 race or ethnicity.

The mean number of workdays missed due to illness, injury, or skipping in the past 30 days increased in a stepwise fashion with increasing AUD severity (no AUD, 1.08 workdays; 95% CI, 1.06-1.10 workdays vs severe AUD, 2.69 workdays; 95% CI, 2.28-3.09 workdays). Respondents with no AUD reported missing 0.84 (95% CI, 0.82-0.86) days per month due to illness or injury and those with severe AUD reported missing 1.67 (95%, 1.38-1.96) days per month (Table 2). Similarly, respondents with no AUD reported skipping 0.24 (95% CI, 0.23-0.25) workdays per month compared with 1.03 (95% CI, 0.84-1.22) workdays skipped per month for those with severe AUD. Total annual absences per individual were estimated to be 13.0 (95% CI, 12.7-13.2) workdays for no AUD, 17.7 (95% CI, 16.4-19.1) workdays for mild AUD, 23.6 (95% CI, 21.5-25.7) workdays for moderate AUD, and 32.3 (95% CI, 27.5-37.0) workdays for severe AUD (Table 2). Although individuals with AUD accounted for 9.3% of the sample, they accounted for 14.1% of all days missed from work. In total, approximately 232 million absences were reported annually among individuals with AUD (mild AUD, 133.3 million; 95% CI, 129.1-137.4 million; moderate AUD, 53.3 million; 95% CI, 50.0-56.7 million; severe AUD, 46.6 million; 95% CI, 43.6-49.6 million) (Table 2).

**Table 2.** Prevalence of Workplace Absenteeism by Alcohol Use Disorder Severity

Characteristic	Alcohol use disorder severity			
	None (n = 98 506 respondents)	Mild (n = 8193 respondents)	Moderate (n = 2432 respondents)	Severe (n = 1570 respondents)
Weighted				
Frequency, No. (95% CI), millions	109.8 (108.6-110.9)	7.5 (7.3-7.7)	2.3 (2.1-2.4)	1.4 (1.4-1.5)
Prevalence, % (95% CI)	90.7 (90.5-91.0)	6.2 (6.0-6.4)	1.9 (1.7-2.0)	1.2 (1.1-1.3)
Missed workdays per person, mean (95% CI), d/mo				
Total	1.08 (1.06-1.10)	1.48 (1.36-1.60)	1.97 (1.79-2.15)	2.69 (2.28-3.09)
Illness/injury	0.84 (0.82-0.86)	1.02 (0.92-1.11)	1.33 (1.18-1.48)	1.67 (1.38-1.96)
Skipping work	0.24 (0.23-0.25)	0.47 (0.41-0.52)	0.66 (0.57-0.74)	1.03 (0.84-1.22)
Total missed workdays per person, mean (95% CI), d/y	13.0 (12.7-13.2)	17.7 (16.4-19.1)	23.6 (21.5-25.7)	32.3 (27.5-37.0)
Weighted				
Total missed workdays/y, No. (95% CI), millions	1422.0 (1407.0-1437.0)	133.3 (129.1-137.4)	53.3 (50.0-56.7)	46.6 (43.6-49.6)
Share of all missed workdays, % (95% CI)	85.6 (84.0-87.2)	8.1 (7.4-8.8)	3.2 (2.9-3.5)	2.8 (2.4-3.2)

Prevalence of missing 3 or more days of work due to illness or injury in the past 30 days also increased in a stepwise fashion with AUD severity (no AUD, 8.8%; 95% CI, 8.5%-9.1%; mild AUD, 11.2%; 95% CI, 10.2%-12.1%; moderate AUD, 14.9%; 95% CI, 13.1%-16.7%; severe AUD, 18.4%; 95% CI, 15.6%-21.2%) (Table 3). The same pattern was observed for prevalence of skipping 3 or more days of work in the past 30 days (no AUD, 2.4%; 95% CI, 2.3%-2.5%; mild AUD, 4.9%; 95% CI, 4.2%-5.6%; moderate AUD, 7.1%; 95% CI, 5.7%-8.5%; severe AUD, 11.8%; 95% CI, 9.7%-13.8%). The same trend was seen with raw prevalence ratios, unadjusted odds ratios, and adjusted odds ratios, with all categories of AUD severity (compared with no AUD) showing significantly increased prevalence of substantial absenteeism, defined as missing either 3 or more days of work in the past 30 days due to illness, injury, or skipping work. Odds ratios were significant for AUD after adjusting for potentially confounding variables of age, sex, race and ethnicity, history of conduct problems, and recent severe psychological distress. Odds of elevated substantial absenteeism increased with AUD severity for both absenteeism outcomes, from mild AUD (OR, 1.15; 95% CI, 1.04-1.28) to severe AUD (OR, 1.71; 95% CI, 1.41-2.07) for missing work due to illness or injury, as well as for skipping work (mild AUD: OR, 1.71; 95% CI, 1.56-1.89; severe AUD: OR, 2.81; 95% CI, 2.36-3.36) (Table 3).

**Table 3.** Prevalence and Odds Ratio Estimates for Full-Time Workers Missing 3 or More Days of Work per Month by Alcohol Use Disorder Severity^a^

AUD severity	Raw frequency (weighted %)	Weighted frequency, No. (95% CI)	Unadjusted (95% CI)		Adjusted OR (95% CI)
			PR	OR	
**Prevalence of missing ≥3 workdays in past 30 d from illness or injury**					
None	9552 (8.8)	9 665 246 (9 369 061-9 961 431)	1 [Reference]	1 [Reference]	1 [Reference]
Mild	1053 (11.2)	839 280 (770 204-908 356)	1.27 (1.16-1.39)	1.30 (1.18-1.44)	1.15 (1.04-1.28)
Moderate	385 (14.9)	336 794 (296 440-377 148)	1.69 (1.50-1.91)	1.81 (1.57-2.09)	1.49 (1.28-1.73)
Severe	289 (18.4)	266 336 (225 752-306 920)	2.09 (1.80-2.44)	2.34 (1.94-2.82)	1.71 (1.41-2.07)
**Prevalence of skipping ≥3 workdays in past 30 d**					
None	2870 (2.4)	2 670 541 (2 544 633-2 796 449)	1 [Reference]	1 [Reference]	1 [Reference]
Mild	473 (4.9)	369 321 (316 599-422 043)	1.87 (1.74-2.02)	2.07 (1.89-2.28)	1.71 (1.56-1.89)
Moderate	184 (7.1)	161 284 (129 757-192 811)	2.41 (2.22-2.63)	2.87 (2.56-3.21)	2.17 (1.88-2.51)
Severe	192 (11.8)	170 977 (139 664-202 290)	3.19 (2.85-3.56)	4.21 (3.59-4.94)	2.81 (2.36-3.36)

Abbreviations: AUD, alcohol use disorder; OR, odds ratio; PR, prevalence ratio.

^a^ Adjusted for age, sex, race and ethnicity, history of conduct problems, and recent severe psychological distress.

## Discussion

Among the adult workforce in the US, 9.3% (almost 11 million full-time workers) met diagnostic criteria for AUD in the past 12 months. There was a clear association between AUD and increased workplace absenteeism; as the severity of AUD increased, so did the number of days missed from work because of illness, injury, or skipping work. Those in the full-time workforce with severe AUD missed an average of 2.69 workdays within the last 30 days compared with 1.08 workdays among people without AUD. When extrapolated to a year, individuals with severe AUD were estimated to miss 32.3 workdays, while those with no AUD were estimated to miss 13.0 workdays. Despite only making up 9.3% of the population, individuals with AUD accounted for 14.1% of all absences.

The workplace can often be the first point of prevention and intervention for individuals with AUD.^22^ Resources such as Employee Assistance Programs have been shown to be generally effective in improving workplace outcomes related to alcohol use.^22^ In recent years, some concern has been raised that these services are increasingly less available to employees, particularly as the economy transitions to more gig-based and other systems of employment that carry fewer employee benefits.^23,24^ The large fraction of work absenteeism associated with AUD in this study is important to public health and to our economy, and provides a strong rationale for increasing investment in strategies to prevent and treat AUD.

### Strengths and Limitations

There are several strengths of this study. First, data were drawn from an annual national survey that remains largely consistent across years, allowing for a larger sample size (over 110 000 respondents) than previous studies of alcohol use and workplace absenteeism.^25,26,27,28,29^ Furthermore, because the NSDUH is designed to be nationally representative, our results are more generalizable than many previous studies that have focused on specific occupations or environments.^30,31,32,33^

A major limitation of previous studies was the difficulty in adequately capturing the specific subpopulation of alcohol users who experienced significant psychosocial consequences.^28,34^ Because drinking alcohol is socially acceptable and common in the US, there is a high prevalence of nonproblematic alcohol use. Studies that use items based on quantity of alcohol intake may obscure the relationship between AUD and workplace absenteeism. Because NSDUH provides a comprehensive assessment of the adverse consequences of alcohol use, our study was able to use current medical diagnostic criteria to define AUD. Defining AUD along the severity continuum is one of the major reasons why we saw clear associations between AUD and workplace absences compared with many previous studies. Additionally, because this study approximated *DSM-5* criteria for AUD, the current system by which individuals with problematic alcohol use are diagnosed, these results are also more likely to be clinically applicable, even relative to prior studies of NSDUH data.^17^

Additionally, because of the comprehensive nature of the NSDUH questionnaire, this study was able to control for multiple other factors that could potentially affect the association between AUD and workplace absenteeism. Two specific variables cited in previous studies as potential confounders were a tendency toward rule-breaking and a recent history of severe psychological distress. Given that we were able to adequately control for these issues, we are confident that neither explains the increased work absenteeism observed among those with AUD.

There are also several limitations to the present study. First, because of the observational nature of this study, no causality can be inferred in the association between AUD and absenteeism. Additionally, because of the limited scope of social questions contained within NSDUH, there were many other important factors impacting absenteeism for which we were unable to control (eg, employment in the service sector, childcare status, medical comorbidities). Because of this, it is possible that the association observed in this study between AUD and absenteeism could be overestimated. Furthermore, our variable of recent psychological distress, an estimate of recent major psychiatric illness, could in some circumstances have a mediating relationship with AUD and absenteeism, something that could also be biasing our results.

Because we used existing data from a national annual survey, we were not able to customize the questions to better characterize the nature of the workplace absenteeism observed. The NSDUH limited types of absences to injury and illness or for skipped days, and it is possible some types of absences were not included in our analysis (eg, staying home to care for a sick family member).

Another limitation is that absenteeism is only 1 negative workplace outcome associated with AUD. There is also a large body of research on the impact of problematic alcohol use on what has been referred to as “presenteeism” in the workplace. In the context of problematic alcohol use, presenteeism has been defined as being physically present at work while the worker is in an impaired state due to drinking, related to intoxication, withdrawal symptoms, and other effects of alcohol.^35^ Research to date suggests that presenteeism related to problematic alcohol use likely contributes to additional economic costs through reduced work efficiency, decreased total work output, and errors on the job.^35^ By examining only physical absences from work, this study likely underestimates the economic cost of AUD in the workplace.

## Conclusions

The question of whether AUD is associated with workplace absenteeism is an important one, and we find a disproportionate prevalence of absenteeism among individuals with AUD. This economic effect is large, and these findings should provide financial incentive for both employers and policy makers alike to invest in support for the prevention of AUD and treatment for individuals with AUD.

## Notes

Supplement. **eAppendix.** Alcohol Use Disorder Coding **eTable 1.** NSDUH Questions that Correspond to *DSM-5* Alcohol Use Disorder (AUD) Criteria **eTable 2.** Concordance Between *DSM-IV* Alcohol Abuse or Dependence and *DSM-5* AUD Within Study Population **eFigure 1.** Study Respondent Inclusion Criteria Flowchart
