# Supplementary material for: Association Between Workplace Absenteeism and Alcohol Use Disorder From the National Survey on Drug Use and Health, 2015-2019
Source: JAMA Netw Open. 2022 Mar 17;5(3):e222954. doi: 10.1001/jamanetworkopen.2022.2954 (PMC8931561; doi:10.1001/jamanetworkopen.2022.2954)
Supplement: Supplement. — eAppendix. Alcohol Use Disorder Coding eTable 1. NSDUH Questions that Correspond to DSM-5 Alcohol Use Disorder (AUD) Criteria eTable 2. Concordance Between DSM-IV Alcohol Abuse or Dependence and DSM-5 AUD Within Study Population eFigure 1. Study Respondent Inclusion Criteria Flowchart [file jamanetwopen-e222954-s001.pdf]

Supplemental Online Content

Parsley IC, Dale AM, Fisher SL, et al. Association between workplace absenteeism and alcohol use disorder from the National Survey on Drug Use and Health, 2015-2019. *JAMA Netw Open.* 2022;5(3):e222954. doi:10.1001/jamanetworkopen.2022.2954

eAppendix. Alcohol Use Disorder Coding

eTable 1. NSDUH Questions that Correspond to *DSM-5* Alcohol Use Disorder (AUD) Criteria

eTable 2. Concordance Between *DSM-IV* Alcohol Abuse or Dependence and *DSM-5* AUD Within Study Population

eFigure 1. Study Respondent Inclusion Criteria Flowchart

This supplemental material has been provided by the authors to give readers additional information about their work.

© 2022 Parsley IC et al. *JAMA Network Open.*

eAppendix. Alcohol Use Disorder (AUD) Coding

National Survey on Drug Use and Health (NSDUH) questions related to alcohol use were developed based on DSM-IV diagnostic criteria[1] and designed to provide diagnoses for past 12-month alcohol abuse and alcohol dependence. DSM-5 criteria,[2] which have been used clinically since 2013, removed the distinction between alcohol abuse and dependence. AUD is now categorized by severity based on the number of 11 possible diagnostic criteria endorsed. The NSDUH has analogous questions for 10 of the 11 DSM-5 AUD criteria. The one DSM-5 criterion without a corresponding NSDUH question is related to craving. Using similar methodology to previous studies,[3,4] we took a graded severity approach similar to the DSM-5 diagnostic approach, classifying respondents who reported 0-1 criteria as not having AUD, those reporting 2-3 criteria as having mild AUD, those reporting 4-5 criteria as having moderate AUD, and those reporting 6 or more criteria as having severe AUD. This method for defining past 12-month AUD severity was chosen for two reasons. First, DSM-5 is the current diagnostic system in use clinically and therefore makes results more directly applicable to current practice. Second, this graded approach can better capture the entire spectrum of problematic behaviors related to alcohol use, a challenge noted in previous studies that used DSM-IV criteria. eTable 1 outlines the NSDUH survey questions that correspond to each individual DSM-5 criterion used to define AUD for this study. There is >95% concordance between DSM-5 AUD and DSM-IV alcohol abuse or dependence (eTable 2). Of those with moderate or severe AUD as defined by DSM-5, 100% also meet the DSM-IV diagnosis for alcohol abuse or dependence. Correspondingly, of those who meet DSM-IV diagnosis for alcohol dependence, 100% also meet DSM-5 diagnosis for AUD.

References

1. American Psychiatric Association. *Diagnostic and Statistical Manual of Mental Disorders.* 4th ed. Washington DC: American Psychiatric Association; 1994.
2. American Psychiatric Association. *Diagnostic and Statistical Manual of Mental Disorders: DSM5.* 5th ed. Washington, D.C.: American Psychiatric Association; 2013.
3. Johnson K, Rigg KK, Hopkins Eyles C. Receiving addiction treatment in the US: Do patient demographics, drug of choice, or substance use disorder severity matter? *Int J Drug Policy.* 2020;75:102583.
4. Mintz CM, Hartz SM, Fisher SL, et al. A cascade of care for alcohol use disorder: Using 2015-2019 National Survey on Drug Use and Health data to identify gaps in past 12-month care. *Alcohol Clin Exp Res.* 2021;45(6):1276-1286.

© 2022 Parsley IC et al. *JAMA Network Open.*

eTable 1: NSDUH questions that correspond to DSM-5 alcohol use disorder (AUD) criteria

DSM-5 Criteria	Variable	Question	Survey Question Text
1. Alcohol is often taken in larger amounts or over a longer period than was intended.	ALCKPLMT	DRALC05	DRAL04: During the past 12 months, did you try to set limits on how often or how much alcohol you would drink?<br>DRALC05 (asked if DRALC04=Yes): Were you able to keep to the limits you set, or did you often drink more than you intended to?<br>1=Usually kept to limits set<br>2=Often drank more than intended *
2. There was a persistent desire or unsuccessful efforts to cut down or control alcohol use.	ALCCUTEV	DRALC09	DRAL08: During the past 12 months, did you want to or try to cut down or stop drinking alcohol?<br>DRALC09 (asked if DRALC08=Yes): During the past 12 months, were you able to cut down or stop drinking alcohol every time you wanted to or tried to?<br>1=Yes<br>2=No *
3. A great deal of time is spent in activities necessary to obtain alcohol, use alcohol, or recover from its effects.	ALCLOTTM	DRALC01	During the past 12 months, was there a month or more when you spent a lot of your time getting or drinking alcohol?<br>1=Yes *<br>2=No
	**OR**		
	ALCGTOVR	DRALC02	During the past 12 months, was there a month or more when you spent a lot of time getting over the effects of the alcohol you drank?<br>1=Yes *<br>2=No
4. Craving, or a strong desire or urge to use alcohol.	N/A	N/A	NOT ASSESSED
5. Recurrent alcohol use resulting in a failure to fulfill major role obligations at work, school, or home.	ALCSERPB	DRALC18	Sometimes people who drink alcohol have serious problems at home, work or school — such as:<br>• Neglecting their children<br>• Missing work or school<br>• Doing a poor job at work or school<br>• Losing a job or dropping out of school<br>During the past 12 months, did drinking alcohol cause you to have serious problems like this either at home, work, or school?<br>1=Yes *<br>2=No

© 2022 Parsley IC et al. *JAMA Network Open.*

DSM-5 Criteria	Variable	Question	Survey Question Text
6. Continued alcohol use despite having persistent or recurrent social or interpersonal problems caused or exacerbated by the effects of opioids	ALCFMCTD	DRALC22	Did you continue to drink alcohol even though you thought your drinking caused problems with family or friends?<br>1=Yes *<br>2=No
7. Important social, occupational, or recreational activities are given up or reduced because of alcohol use.	ALCLSACT	DRALC17	This question is about important activities such as working, going to school, taking care of children, doing fun things such as hobbies and sports, and spending time with friends and family.<br>During the past 12 months, did drinking alcohol cause you to give up or spend less time doing these types of important activities?<br>1=Yes *<br>2=No
8: Recurrent alcohol use in situations in which it is physically hazardous.	ALCPDANG	DRALC19	During the past 12 months, did you regularly drink alcohol and then do something where being drunk might have put you in physical danger?<br>1=Yes *<br>2=No
9. Continued alcohol use despite knowledge of having a persistent or recurrent physical or psychological problem that is likely to have been caused or exacerbated by the substance.	ALCEMCTD	DRALC14	Did you continue to drink alcohol even though you thought drinking was causing you to have problems with your emotions, nerves, or mental health?<br>1=Yes *<br>2=No
	**OR**		
	ALCPHCTD	DRALC16	DRALC15: During the past 12 months, did you have any physical health problems that were probably caused or made worse by drinking alcohol?<br>DRALC16 (asked if DRALC15=Yes):<br>Did you continue to drink alcohol even though you thought drinking was causing you to have physical problems?<br>1=Yes *<br>2=No

© 2022 Parsley IC et al. *JAMA Network Open.*

DSM-5 Criteria	Variable	Question	Survey Question Text
10: Tolerance, as defined by either of the following: a) A need for markedly increased amounts of alcohol to achieve intoxication or desired effect; b) A markedly diminished effect with continued use of the same amount of alcohol.	ALCNDMOR	DRALC06	During the past 12 months, did you need to drink more alcohol than you used to in order to get the effect you wanted?<br>*1=Yes<br>2=No
	**OR**		
	ALCLSEFX	DRALC07	During the past 12 months, did you notice that drinking the same amount of alcohol had less effect on you than it used to?<br>*1=Yes<br>2=No
11: Withdrawal, as manifested by either of the following: a) The characteristic withdrawal syndrome for alcohol; b) Alcohol (or a closely related substance, such as a benzodiazepine) is taken to relieve or avoid withdrawal symptoms.	ALCWD2SX	DRALC11	Please look at the symptoms listed below. During the past 12 months, did you have 2 or more of these symptoms after you cut back or stopped drinking alcohol?<br>• Sweating or feeling that your heart was beating fast<br>• Having your hands tremble<br>• Having trouble sleeping<br>• Vomiting or feeling nauseous<br>• Seeing, hearing, or feeling things that weren’t really there<br>• Feeling like you couldn’t sit still<br>• Feeling anxious<br>• Having seizures or fits<br>*1=Yes<br>2=No

The sum of positive responses, denoted by * in the table above, provided a score of 0 to 10. All missing data was categorized as NO responses. Severity was then subsequently categorized as below:
0-1 = No AUD
2-3 = Mild AUD
4-5 = Moderate AUD
6 or more = Severe AUD

© 2022 Parsley IC et al. *JAMA Network Open.*

eTable 2. Concordance between DSM-IV alcohol abuse or dependence and DSM-5 AUD within study population.

	DSM-5 AUD			
	No	Mild AUD	Moderate AUD	Severe AUD
	Weighted frequency (weighted %)	Weighted frequency (weighted %)	Weighted frequency (weighted %)	Weighted frequency (weighted %)
**DSM-IV alcohol abuse or dependence**				
Yes	1,351,231 (1.1%)	3,419,705 (2.8%)	2,259,939 (1.9%)	1,444,981 (1.2%)
No	108,409,881 (89.6%)	4,091,244 (3.4%)	0 (0.0%)	0 (0.0%)

DSM=Diagnostic and Statistical Manual of Mental Disorders; AUD=alcohol use disorder; NSDUH= National Survey on Drug Use and Health; SE= standard error. Weighted percent is rounded to one decimal place.

© 2022 Parsley IC et al. *JAMA Network Open.*

eFigure 1: Study respondent inclusion criteria flow chart

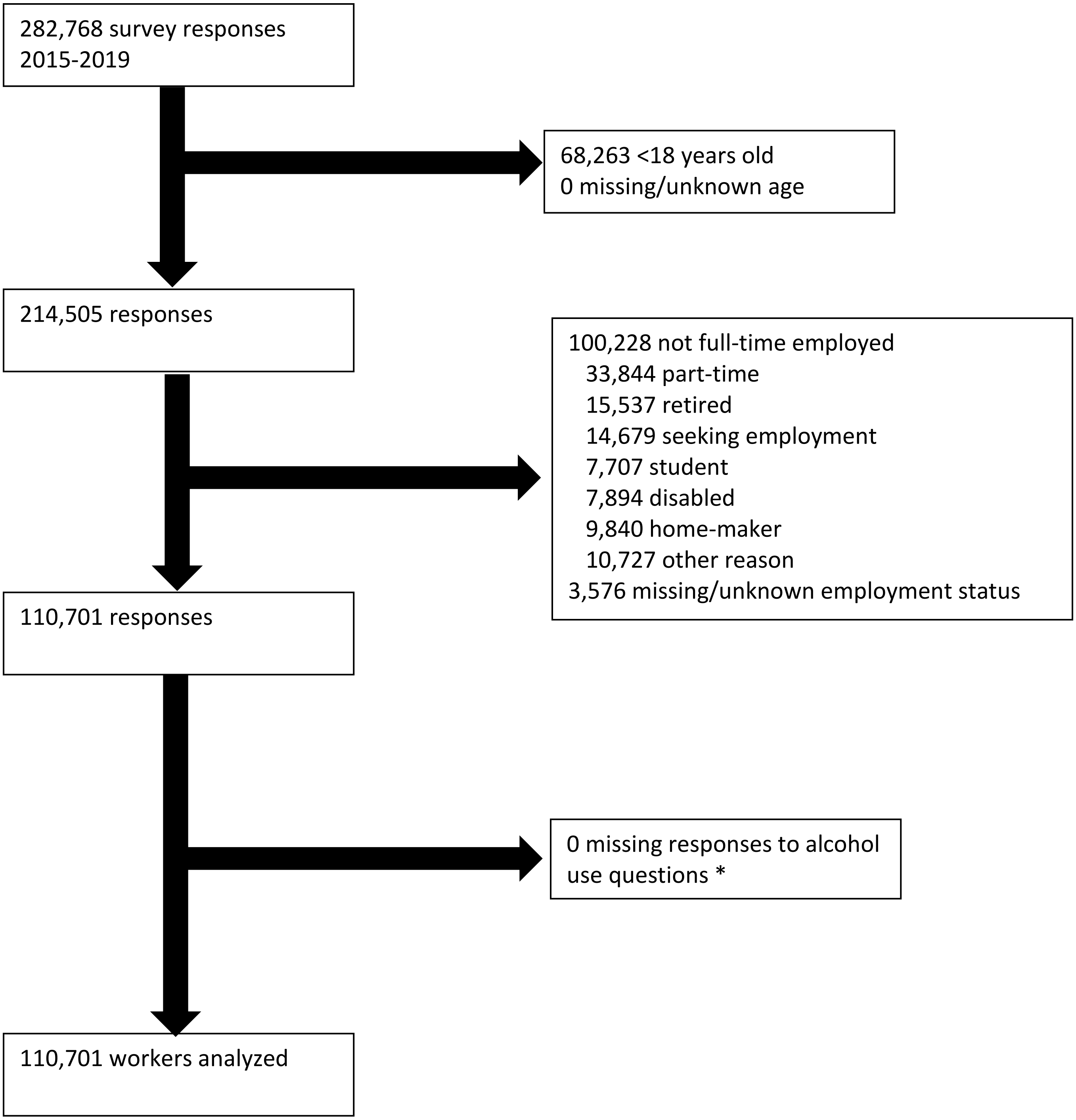

© 2022 Parsley IC et al. *JAMA Network Open.*

*There were no missing responses for AUD severity, as this variable was generated as a sum of responses to multiple questions. Missing responses for these individual alcohol use questions were assumed to be negative (e.g. a missing response to “During the past 12 months, did you need to drink more alcohol than you used to in order to get the effect you wanted?” was coded as “no”).

© 2022 Parsley IC et al. *JAMA Network Open.*
